# COVID-19 and oil market risks: Evidence from new datasets

**DOI:** 10.1016/j.mex.2023.102008

**Published:** 2023-01-07

**Authors:** Afees A. Salisu, Yinka S. Hammed

**Affiliations:** a Centre for Econometrics and Applied Research, Ibadan, Nigeria; b Department of Economics, University of Pretoria, Private Bag X20, Hatfield, 0028, South Africa

**Keywords:** Oil prices, Oil market risk, COVID-19, Predictability, Feasible Quasi-GLS Estimator, Clark and West [3]

## Abstract

We evaluate the predictive value of the newly constructed six COVID-19 indices for oil market risks from 31st December, 2019 (when COVID-19 started) to 28th December, 2021. We show that, on average, higher values of the COVID-19 indices appear to have heightened oil market risks albeit with the converse for Vaccine index regardless of the choice of oil price proxy. The predictive value of the indices is sustained over multiple out-of-sample forecasts and we attribute the outcome to the increased uncertainties associated with the pandemic. Therefore, measures aimed at mitigating these uncertainties can help moderate the oil market risks.

• Testing the predictive value of the newly constructed COVID-19 measures for the out-of-sample forecasting of oil market risks.
• Increased uncertainties associated with the pandemic tend to raise the level of oil market risks.
• Measures aimed at mitigating these uncertainties can help moderate the oil market risks.

## Graphical abstract

**Figure:**
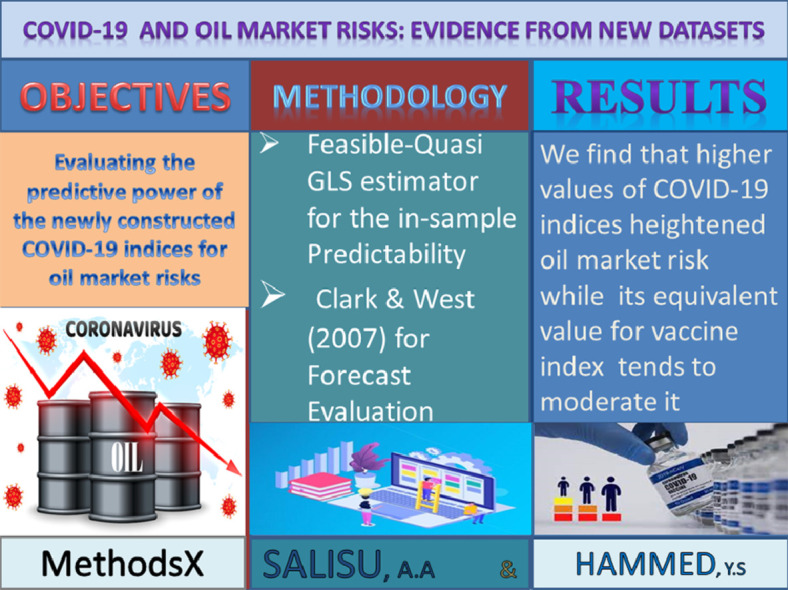

Specifications table

**Table:** 

Subject area:	Economics and Finance
More specific subject area:	Energy Economics
Name of your method:	Feasible Quasi-GLS Estimator, Clark and West [3]
Name and reference of original method:	Not Applicable
Resource availability:	Not Applicable

**Method details**

## Introduction

The connection between COVID-19 pandemic and crude oil market is well-established in the literature (see [4,[6], [7], [8],^13^,^18^,^23^], among others). However, our interest to revisit the nexus is motivated by the newly constructed six COVID-19 indices by Narayan, Iyke and Sharma [12]. These indices remain the most comprehensive description of the pandemic as they cover various dimensions of the pandemic namely medical, travel, uncertainty, vaccines, COVID and aggregate COVID and are drawn from relatively exhaustive dictionary of 327 words. Thus, the main objective of this study is to evaluate the predictive value of these indices for crude oil market volatility since this market is considered to be among the worst hit by the pandemic.^1^ As long as the issue of the pandemic remains a topical issue globally, the need to provide robust evidence-based outcomes that strengthen our understanding of the severity of the uncertainties associated with the pandemic becomes even more compelling. Investors and policy constantly seek for such technical support to guide them when taking both short and long term decisions.

Our analyses include both the in-sample and out-of-sample evaluations as the former cannot translate into improved performance of the latter. Therefore, both forecast analyses are necessary to establish the predictability of the considered COVID-19 indices. The forecast procedure follows the approach of Westerlund and Narayan [25,26] as a way to control for the presence of persistence effect and endogeneity problem which are typical of most time series including those examined in this study. We also evaluate the robustness of our findings in the following ways. First, we test the predictability of all the six COVID-19 indices by Narayan et al. [12] rather than limiting the analyses to their aggregate COVID-19 index. This enables us to see the peculiarities of the five sub-indices as some of them tend to exhibit a pattern different from the aggregate index as highlighted in the next paragraph and in the results section of the study. Second, we consider two alternative oil price proxies namely West Texas Intermediate and Brent crude oil prices. These two robustness tests offer sufficient information about the predictive value of the newly constructed COVID-19 indices and whether the choice of COVID-19 index and oil price proxy matters for any relationship to be established between them. In addition, we also test the robustness of the findings of Salisu, Tchankam, and Adediran [22] which appears to be the first paper to test the predictability of these indices albeit with a focus on stock market. In other words, we want to see whether the observed predictive value of the indices for stock market can be extended to the crude oil market.

Our findings can be summarized as follows. First, out of all the set of six indices considered, the aggregate index and vaccine index are consistently found to have significant relationship with oil market risks regardless of the choice of oil price proxy. However, while the relationship with aggregate COVID index is positive, that of vaccine index is negative. In other words, increased uncertainties due to the pandemic are capable of raining the level of oil market risks while measures taken to reduce the uncertainties in terms of increased Vaccines tend to moderate oil market risks. Second, the out-of-sample forecast evaluation of the indices shows some improved level of forecast accuracy of crude oil market risks relative to the benchmark models that ignore the indices. Therefore, as long as the pandemic lingers, accounting for it in the predictive model of crude oil market risk, among other predictors, is crucial for improved forecast accuracy.

## Data and preliminary analysis

The data for this study contains some set of newly generated indices as measures of COVID-19 pandemics. The indices are generated for: COVID, medical, travel, uncertainty, vaccines and aggregate COVID indices (see Narayan, Iyke and Sharma, [12] for technical details of the indices).^2^ The frequency of these data is daily (a 5-day per week) and runs from 31st December, 2019 to 28th December, 2021. Our data for crude oil (both Brent and West Texas Intermediate (WTI)) is sourced from investing.com (https://investing.com/markets/), and its scope covers the same period as for the COVID-19 indices. For these variables (crude oil prices), we used transformed series by computing their realized volatilities. For the latter, we use a 20 day rolling window (based on the trading days per month) and 252 annual trading days. The descriptive statistics of our variables are presented in Table 1 and they cover statistics such as the mean, dispersion, skewness and kurtosis. Out of all the six indices for COVID-19, uncertainty index has the highest average value (45.4) and is mostly dispersed (having dispersion value of 16.9) while vaccine index has the lowest average value (25.6). All the data, except the data for aggregate covid-19 index, have long tail to the right (i.e. they are positively skewed), while at the same time they are all leptokurtic (the value for kurtosis being more than 3). For the oil market risks, the average value of realized volatility for WTI is higher (about 90.6) than that of Brent, more dispersed, skewed more towards the right and has a very high-hump shape curve (having kurtosis value of 21.8). Also, our data are more persistent and there is presence of serial correlation. In addition, the ARCH effect is equally noticed for all of them except for oil market risk variables (realized volatility for Brent and WTI prices).

**Table 1:** Descriptive Statistics.

Statistics	A_CovIndex	CovIndex	MedIndex	TravIndex	UncIndex	VacIndex	RV_WTI	RV_BRENT
Mean	43.668	33.365	37.850	34.111	45.443	25.559	90.661	43.533
Std. Dev.	16.156	14.369	14.254	13.337	16.929	15.738	227.86	33.775
Skewness	-0.1762	0.7193	0.2401	0.7939	0.0103	1.4959	4.4922	2.1508
Kurtosis	4.3921	6.2199	5.2071	7.0992	3.9705	5.8478	21.758	6.6052
Persistence	0.0004^b^	0.4799^a^	0.1935^a^	0.1501^a^	-0.1067^a^	0.6068^a^	0.9713^a^	0.9889^a^
Serial correl.								
Ω(2)	31.698^a^	134.17^a^	65.637^a^	38.352^a^	32.422^a^	112.59a	10.158^a^	8.8258^a^
Ω(5)	186.66^a^	226.94^a^	208.71^a^	112.44^a^	162.99^a^	140.44^a^	4.3272^a^	3.9127^a^
Ω(10)	110.77	152.47^a^	127.81^a^	62.195^a^	90.129^a^	86.589^a^	2.4289^a^	3.2109^a^
								
Cond. Hetero.								
ARCH (2)	1.5816	2.0249	0.8243	0.8450	2.9295^b^	10.8738^a^	0.3571	0.3086
ARCH (5)	186.66^a^	150.33^a^	113.35^a^	43.912^a^	147.61^a^	78.2185^a^	0.1427	0.1219
ARCH (10)	110.77^a^	96.051^a^	75.231^a^	28.162^a^	76.757^a^	46.8446^a^	0.0712	0.5048
Observations	503	503	503	503	503	503	503	503

*Note:* The summaries are done for 503 observation points, where ARCH (#) is for the conditional heteroscedasticity test, Ω(#) is for the serial correlation test with squared residuals. The tests are conducted at different lags for robustness where #= 2, 5, 10. The oil market risk proxies are represented as RV_WTI and RV_Brent obtained from the realized volatilities of WTI and Brent crude oil prices, respectively. The persistence test involves regressing the dependent variable on its first lag and a constant term while the degree of persistence is derived from the coefficient of the first lag. The closer the coefficient to 1, the higher the degree of persistence while the closer it is to zero, the lower the degree of persistence.^a^, ^b^ and ^c^ indicate statistical significance at 1%, 5% and 10% levels respectively.

**Fig. 1:**
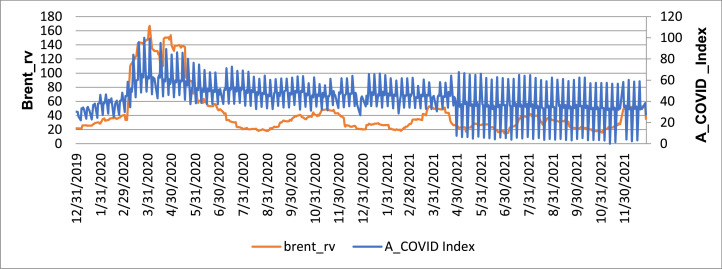
Co-movement between realized volatility of Brent crude oil price (Brent_rv) and Aggregate COVID index (A_COVID_Index).

**Fig. 2:**
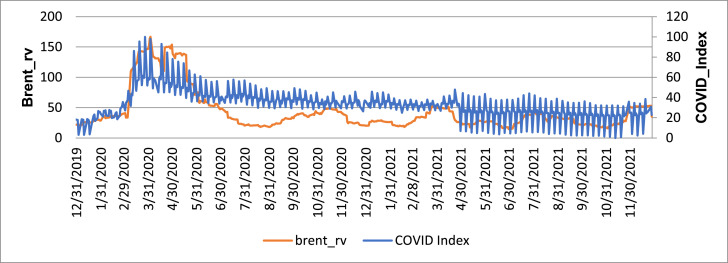
Co-movement between realized volatility of Brent crude oil price (Brent_rv) and COVID index.

**Fig. 3:**
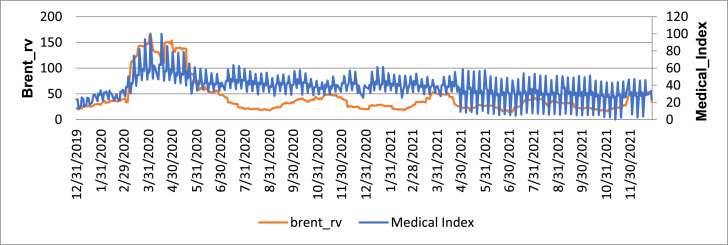
Co-movement between realized volatility of Brent crude oil price (Brent_rv) and medical index.

**Fig. 4:**
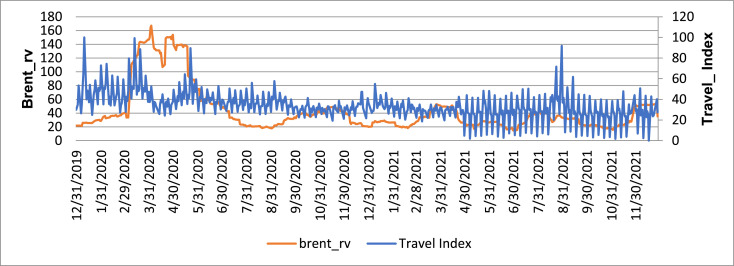
Co-movement between realized volatility of Brent crude oil price (Brent_rv) and travel index.

**Fig. 5:**
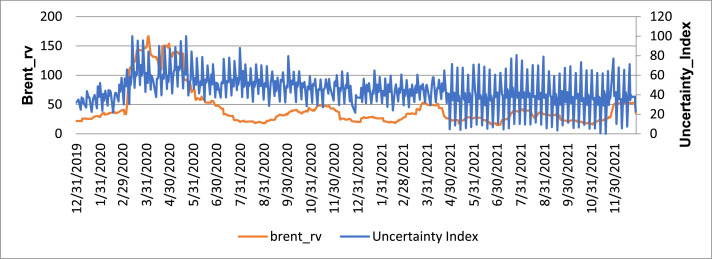
Co-movement between realized volatility of Brent crude oil price (Brent_rv) and uncertainty index.

**Fig. 6:**
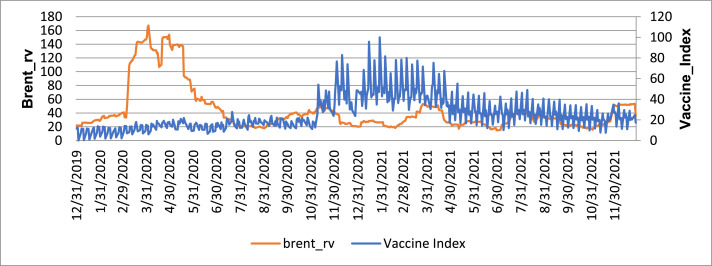
Co-movement between realized volatility of Brent crude oil price (Brent_rv) and vaccine index.

**Fig. A1:**
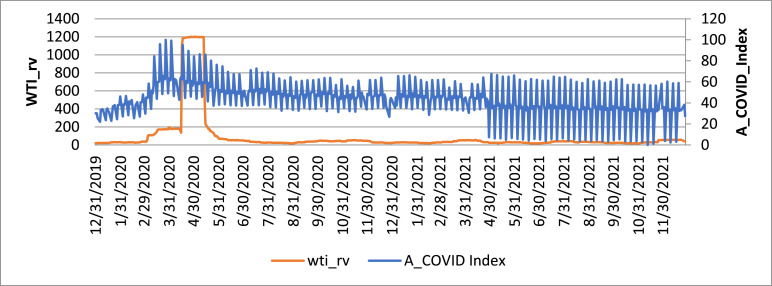
Co-movement between realized volatility of WTI crude oil price (wti_rv) and Aggregate COVID index.

**Fig. A6:**
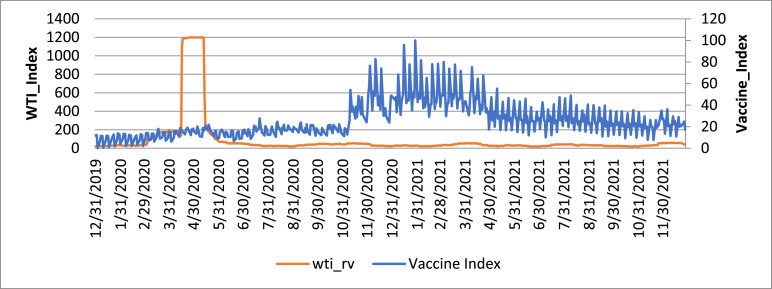
Co-movement between realized volatility of WTI crude oil price (wti_rv) and vaccine index.

Figs. 1 to 6 show the trends in the realized volatility of Brent crude oil price and the respective COVID-based indices (those for the WTI crude oil price are presented in the Appendix in Figs. A1 to A6). The co-movement in these series somewhat suggests a positive relationship between them except for Travel and Vaccine indices. We further assess this relation using an empirical model which is explained in the section that follows.

## Methodology

The methodology for this study is built on Arbitrage Pricing Theory, given our interest in constructing predictive model for oil market risks where we attribute these risks to COVID-19 pandemic. Hence, by way of replication, we singly introduce newly constructed COVID-19 indices of Narayan et al. [12] as the predictors in the model. We strictly follow Westerlund and Narayan [25,26] in our approach to control for special features that our data exhibit such as heteroscedasticity and persistence effects which have already been established in our preliminary analysis. Owing to this, we specify our predictive model as follows:

(1) $$OIL_{t}=\alpha +\rho OIL_{t-1}+\beta_{1}COVID_{t-1}+\gamma_{1}\left(COVID_{t}-\tau_{1}COVID_{t-1}\right)+\phi^{\prime }Z+\varepsilon_{t}$$

where $OIL_{t}$ is the indicator of oil risks, derived by taking natural logarithm of the realized volatility for the oil price benchmarks; $COVID_{t}$ denotes a particular COVID-based index (captured distinctly for each of the indices); α is the intercept; $\beta_{1}$ is the bias-corrected predictability coefficient for the COVID-based index while the term $\gamma_{1}\left(COVID_{t}-\tau_{1}COVID_{t-1}\right)$ is included to account for any inherent persistence effect as well as endogeneity bias^3^ in the model, and $\varepsilon_{t}$ is a zero mean idiosyncratic error term. The introduction of first lag of $OIL_{t}$ as an additional regressor in Eq. (1) is to correct for volatility persistence as evident in Table 1. Hence, the null hypothesis of no predictability $H_{0}:\beta_{1}=0$ between COVID indices ($COVID_{t}$) and oil market risk ($OIL_{t}$) is tested against the alternative hypothesis of the presence of predictability$H_{1}:\beta_{1}\neq 0$. We also pre-weight our data as a way to control for conditional heteroscedasticity effect with the term $1/{\hat{\sigma }}_{\varepsilon }$in Eq. (1) and subsequently estimate the resulting equation with the Ordinary Least Squares (OLS). This can be described in this case as feasible quasi-GLS estimator (see [25,26]). For robustness, both the Brent and West Texas Intermediate crude oil markets are considered and thus, Eq. (1) is presented as Models 1 and 2 in all the results tables where Models 1 and 2 denote the COVID-based predictive models under the realized volatility of Brent and WTI crude oil price returns, respectively. Note that we also account for structural breaks in the predictability analysis using the Bai & Perron [1] test. This test accommodates up to five structural breaks and deals with structural changes in linear regressions unlike Narayan and Popp [10] test which is series based and does not necessarily account for structural changes in economic relationships. An alternative unit root test that also examines structural changes in economic relationships is the one proposed by Ditzen, Karavias, and Westerlund [5] which has two variants, one for pure time series and the other for panel data. Interestingly, the variant for pure time series equally implements the methods developed by Bai and Perron (1998) which is essentially what is applied in this study. In addition, the gold market risk is included as a control variable given the market hedging relationship with that of crude oil (see [24]). All the additional (control) variables with their corresponding parameters are captured with $\phi^{\prime }Z$ in Eq. (1).

Finally, we evaluate the out-of-sample forecast performance of the COVID-based (unrestricted) model relative to a benchmark (restricted) model where we consider two variants namely random walk with and without drift. The random walk model is a standard benchmark model in time series forecasting as most financial and economic series typically exhibit random walk properties (see for example, [9]) and therefore modellers often consider any economic model that beats this benchmark model as suitable for forecasting. Since both the restricted and the unrestricted models are nested, we use the Clark & West [3] test and opt for the 80:20 data split option for the in-sample predictability and out-of-sample forecast evaluation, respectively. The latter is done for multiple forecast horizons covering, 10, 20, and 30-days ahead forecast horizons under a rolling window framework that allows for some time-variation in the forecast analyses.

## Results and discussion

Table 2 presents the result for the predictability of our variables of focus which are the newly constructed COVID-19 indices by Narayan et al. [12] to verify their predictive power on the realized volatility of oil price (which we term as oil market risks). As previously stated, full data sample is used for the estimate of predictability analysis while the split of 80% of data for in-sample analysis and 20% for out-of-sample forecast estimation is adopted. In our result, we find direct relationship between oil risks and aggregate COVID index and COVID-index in both models and indirect with vaccine index. As for other indices, the relationship is alternating as either direct or otherwise between the two models. The significance of the relationship between the indices and the oil market risk is found to be significant in model 2 (except for the uncertainty index) and in model 1 (except for both COVID and medical indices). By implication, oil market risks rise with increase in both aggregate COVID index and COVID index and fall with increase in vaccine index, while the implication is case-specific for other indices with respect to the two alternative models. For instance, medical index (in model 1 where we use Brent as the proxy for oil price) and travel, and uncertainty indices (in model 2 where we use WTI as the proxy for oil price) exhibit inverse relationship with oil price realized volatility. If we restrict ourselves to the results from aggregate COVID index and COVID index which are invariably the same, it will suggest that higher levels of COVID-19 cases will result in rising oil market risks while higher levels of Vaccine index tend to moderate the oil market risk. Put differently, while the uncertainties associated with the pandemic can deepen the oil market risks, improvements in the measures taken to reduce these uncertainties through increased distribution of vaccines can help moderate the risks.

**Table 2:** Predictability results.

	Model 1	Model 2
A_COVID Index	0.0049^⁎⁎^ [2.4975]	0.0207^⁎⁎⁎^ [5.7729]
COVID Index	0.0002 [0.0750]	0.0311^⁎⁎⁎^ [8.2918]
Medical Index	-0.0025 [-1.0628]	0.0366^⁎⁎⁎^ [10.808]
Travel Index	0.0132^⁎⁎⁎^ [8.6211]	-0.0117^⁎⁎⁎^ [-3.0101]
Uncertainty Index	0.0092^⁎⁎⁎^ [5.2449]	-0.0004 [-0.1060]
Vaccine Index	-0.0054^⁎⁎⁎^ [-3.6025]	-0.0072^⁎⁎^ [-2.2809]

*Note:* The results presented in the table are for the predictability of the six different COVID indices under alternative oil price proxies where Models 1 and 2 are respectively for the realized volatilities of Brent and WTI crude oil price returns.^⁎⁎⁎^, ^⁎⁎^ and * indicate statistical significance at 10%, 5% and 1% levels respectively.Results in square brackets [] are the t-statistics.

Also, accuracy of in-sample predictability analysis does not suggest that an out-of-sample analysis will also be accurate. Owing to this, we carry out an out-of-sample forecast evaluation for oil market risk predictability. Essentially, we use a formal pairwise comparison tool involving Clark and West [3] test.^4^ The rule in this analysis is that the estimated statistics from the C-W test must be positive and significant to adjudge our model as better when compared to the benchmark. We show in Table 3 and 4 (for random walk model without drift as the benchmark model andfor random walk model with drift as the benchmark model, respectively) the forecast evaluation results for each of the COVID-based predictive models and the benchmark models and we are able to confirm the superiority of the COVID-based predictive models over the benchmark models involving random walk with and without drift. All the test statistics are statistically significant and in a way suggest that the inclusion of the information contents of the COVID along with other important predictors in the predictive model of oil market risk will enhance forecast accuracy.^5^ In other words, accounting for COVID–related uncertainties in the predictive model of crude oil market risk is crucial as long as the pandemic lingers.

**Table 3:** Forecast Evaluation using C-W test where Random Walk without drift is the benchmark model.

COVID Indices	In-Sample		Out-of-Sample					
			h=10		h=20		h=30	
	Model 1	Model 2	Model 1	Model 2	Model 1	Model 2	Model 1	Model 2
A_COVID Index	7.3298^a^	8.0991^a^	7.3159^a^	8.1403^a^	7.3084^a^	8.2036^a^	7.3061^a^	8.2930^a^
COVID Index	7.8740^a^	8.2061^a^	7.8537^a^	8.2412^a^	7.8647^a^	8.3051^a^	7.8861^a^	8.3960^a^
Medical Index	8.2553^a^	7.8682^a^	8.2294^a^	7.9057^a^	8.2194^a^	7.9717^a^	8.2266^a^	8.0490^a^
Travel Index	6.8616^a^	7.4623^a^	6.8479^a^	7.4907^a^	6.8583^a^	7.5084^a^	6.8396^a^	7.5546^a^
Uncertainty Index	8.6210^a^	7.7833^a^	8.5970^a^	7.8116^a^	8.6063^a^	7.8297^a^	8.6230^a^	7.8839^a^
Vaccine Index	9.1728^a^	7.6180^a^	9.1343^a^	7.6458^a^	9.1398^a^	7.6544^a^	9.1613^a^	7.6946^a^

*Note:* The results presented in the table are for the forecast evaluation of the (COVID-based) model and the restricted (random walk without drift) model using the Clark and West (2007) [C-W] test. Models 1 and 2 are respectively for the realized volatilities of Brent and WTI crude oil price returns which are singly compared with the benchmark model.The t-statistics are presented for this purpose and ^a^, ^b^ and ^c^ indicate statistical significance at 1%, 5% and 10% levels, respectively.Our proposed (COVID-based) predictive model has better forecast performance than the benchmark model when the t-statistic is positive and statistically significant, otherwise, the benchmark model is considered superior.

**Table 4:** Forecast Evaluation using C-W test where Random Walk with drift is the benchmark model.

COVID Indices	In-Sample		Out-of-Sample					
			h=10		h=20		h=30	
	Model 1	Model 2	Model 1	Model 2	Model 1	Model 2	Model 1	Model 2
A_COVID Index	7.7253^a^	8.2054^a^	9.8493^a^	8.7403^a^	9.8466^a^	8.8309^a^	9.8531^a^	8.9473^a^
COVID Index	8.4178^a^	8.3063^a^	10.374^a^	8.7907^a^	10.413^a^	8.8822^a^	10.469^a^	9.0006^a^
Medical Index	8.8049^a^	8.0002^a^	11.083^a^	8.5315^a^	11.085^a^	8.6262^a^	11.117^a^	8.7296^a^
Travel Index	7.4091^a^	7.5780^a^	9.8775^a^	7.7138^a^	9.8961^a^	7.7454^a^	9.8739^a^	7.8097^a^
Uncertainty Index	9.1438^a^	7.8938^a^	11.298^a^	8.2244^a^	11.337^a^	8.2570^a^	11.387^a^	8.3312^a^
Vaccine Index	9.6320^a^	7.7289^a^	11.434^a^	8.0090^a^	11.469^a^	8.0288^a^	11.530^a^	8.0859^a^

*Note:* Except that this statistics is for random walk with drift, other information is as contained in note Table 5.

## Conclusion

In this study, we examine the predictability of the newly constructed COVID-19 indices by Narayan et al. [12] for oil market risk (realized volatility of oil prices for Brent and WTI). We argue from the perspective of the Arbitrage Pricing Theory which suggests that asset returns are susceptible to different risk factors and therefore we attribute the uncertainties due to the COVID-19 pandemic a form of systemic risk to the crude oil market. Thus, we evaluate both the in-sample and out-of-sample predictability of these new datasets for COVID-19 while controlling structural breaks and gold market risk.

We find that out of all the six indices for the COVID-19 as constructed by Narayan et al. [12], only aggregate COVID-index and vaccine index show consistent in-sample predictability for the two oil price proxies. However, while aggregate COVID-19 has a positive relationship, vaccine index is negative. By implication, the associated risks with oil prices rise with rising aggregate COVID index and fall with rising vaccine index. Thus, while COVID-related uncertainties are capable of raising oil market risks, more awareness about the possibility of vaccines can help moderate the risks. Therefore, the current efforts by the international community and individual governments towards ensuring a wider vaccination coverage should be intensified and sustained.

## Declaration of Competing Interest

The authors declare that they have no known competing financial interests or personal relationships that could have appeared to influence the work reported in this paper.

## Figures and Tables

**Fig. A2:**
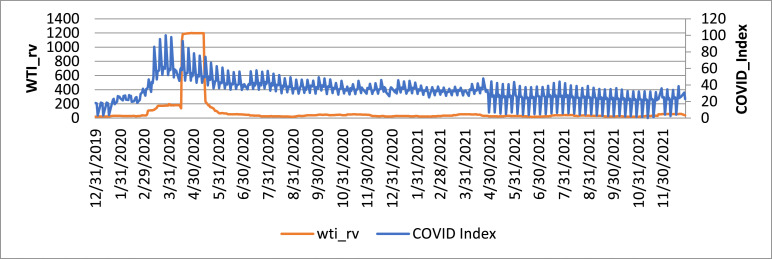
Co-movement between realized volatility of WTI crude oil price (wti_rv) and COVID index.

**Fig. A3:**
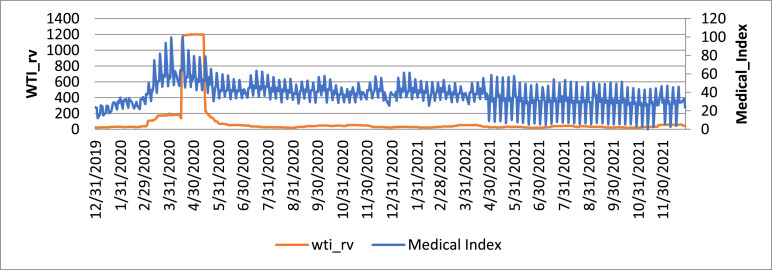
Co-movement between realized volatility of WTI crude oil price (wti_rv) and medical index.

**Fig. A4:**
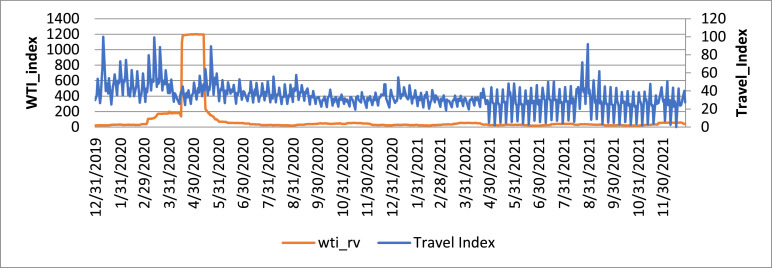
Co-movement between realized volatility of WTI crude oil price (wti_rv) and travel index.

**Fig. A5:**
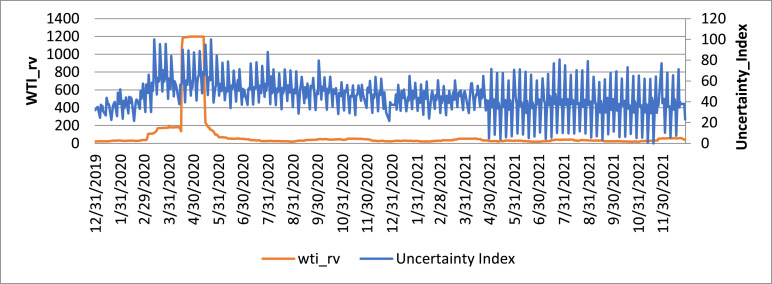
Co-movement between realized volatility of WTI crude oil price (wti_rv) and uncertainty index.

## Data Availability

Data will be made available on request.

## Appendix

Figs. A1, A2, A3, A4, A5, A6

## Footnotes

1 Other alternative measures of COVID-19 pandemic involve the COVID-19-induced fear index by Salisu and Akanni [20] and the index for infectious diseases by Baker et al. [2]. The predictability of these indices has also been evaluated for crude oil market, among other commodity markets (see [20,21]). Also recently, Salisu, Tchankam, and Adediran, [22] use the newly constructed indices to predict the stock markets of emerging economies using a panel data framework and the evidence confirms the high predictive value of some of them particularly the one that incorporates all the other five variants (aggregate COVID).

2 Please note that the data presented in the published article of Narayan et al. [12] only ends at 4/28/2021. However, an updated version of the data that ends at 12/28/2021 was provided by Prof. Paresh Narayan and we are indeed grateful for this kind gesture. The updated data enables us to capture more recent dynamics associated with the pandemic.

3 As oil price is typically considered as an exogenous factor when modelling the inherent dynamics in most financial and economics series (see for example, [11,[14], [15], [16], [17],^19^]), specifying it as an endogenous variable as done in this study is expected to introduce some endogeneity bias which has to be dealt with in the estimation process.

4 The forecast evaluation technique is limited to the Clark and West [3] test for want of space and more importantly due to the fact that it is particularly suitable for nested models which is the case in this study.

5 Other relevant forecast evaluation results such as the individual RMSE values for all the predictive models including the benchmark models used in the analysis of the Clark and West [3] test are available upon request.
